# Author Correction: Rurality and relative poverty drive acquisition of a stable and diverse gut microbiome in early childhood in a non-industrialized setting

**DOI:** 10.1038/s41598-025-95200-w

**Published:** 2025-04-01

**Authors:** Victor Seco-Hidalgo, Adam A. Witney, Martha E. Chico, Maritza Vaca, Andrea Arevalo, Alexander J. Schuyler, Thomas A. E. Platts-Mills, Irina Chis Ster, Philip J. Cooper

**Affiliations:** 1 Institute of Infection and Immunity, St George’s University of London, London, SW17 0RE UK; 2 Escuela de Medicine, Universidad Internacional del Ecuador, Quito, Ecuador; 3 Fundación Ecuatoriana Para La Investigación en Salud, Quito, Ecuador; 4 Division of Allergy & Clinical Immunology, University of Virginia, Charlottesville, VA USA

Correction to: *Scientific Reports* 10.1038/s41598-025-89224-5, published online 15 February 2025

The Acknowledgements section in the original version of this Article was incomplete, where a project funding number was omitted.

“We thank the ECUAVIDA study team for their dedicated work and the cohort mothers and children for their enthusiastic participation. We acknowledge also the support of the Directors and staff of the Hospital ‘Padre Alberto Buffoni’ in Quininde, Esmeraldas Province.”

now reads:

"We thank the ECUAVIDA study team for their dedicated work and the cohort mothers and children for their enthusiastic participation. We acknowledge also the support of the Directors and staff of the Hospital ‘Padre Alberto Buffoni’ in Quininde, Esmeraldas Province. The study was funded by Wellcome Trust (088862/Z/09/Z) and NIH (RO1-AI-20565) grants.”

The original Article has been corrected.

