# Supplementary material for: Rurality and relative poverty drive acquisition of a stable and diverse gut microbiome in early childhood in a non-industrialized setting
Source: Sci Rep. 2025 Feb 15;15:5601. doi: 10.1038/s41598-025-89224-5 (PMC11830098; doi:10.1038/s41598-025-89224-5)
Supplement: Supplementary file 3 — Supplementary Information 3. [file 41598_2025_89224_MOESM3_ESM.docx]

**Rurality and relative poverty drive acquisition of a stable and diverse gut microbiome in early childhood in a non-industrialized setting**

Victor Seco-Hidalgo^1^, Adam A Witney^1^, Martha E Chico^3^, Maritza Vaca^3^, Andrea Arevalo^3^, Alexander J Schuyler^4^, Thomas AE Platts-Mills^4^, Irina Chis Ster^1^*, Philip J Cooper^1,2,3^*

*Contributed equally

1. Institute of Infection and Immunity, St George’s University of London, London, UK

2. Escuela de Medicine, Universidad Internacional del Ecuador, Quito, Ecuador

3. Fundación Ecuatoriana Para la Investigación en Salud, Quito, Ecuador

4. Division of Allergy & Clinical Immunology, University of Virginia, Charlottesville, Virginia, USA

*Correspondence:

Philip Cooper,

Institute of Infection and Immunity,

St George’s University of London,

London SW17 0RE,

United Kingdom

[pcooper@sgul.ac.uk](mailto:pcooper@sgul.ac.uk)

**Supplementary information**

|  | **Month (95% CI)** | **Log values (95% CI)** | **Original scale (95% CI)** |
| --- | --- | --- | --- |
| **Chao** | 45 (41-49) | 6.7 (6.6-6.9) | 830 (732-937) |
| **Shannon** | 45 (42-48) | 1.2 (1.15-1.27) | 3.33(3.14-3.51) |
| **InvSimpson** | 43 (39-48) | 2.3 (2.2-2.5) | 10.4 (5.6-15.3) |

Table S1. Estimated peak values for each alpha metric and age (months) at which they occurred. The values are estimated after fitting a mixed model to the longitudinal observations representing age-specific scores and can be visualized as average trajectories provided in Figure 1 (bold black line).

| **Variables** | **Summary/**  **Category** | **Values/**  **Frequencies** | **Age-adjusted effects**  **CHAO** | | | | **Age-adjusted effects**  **SHANNON** | | | | **Age-adjusted effects**  **INVSIMPSON** | | | | **Age-adjusted effects**  **BETA** | | | |
| --- | --- | --- | --- | --- | --- | --- | --- | --- | --- | --- | --- | --- | --- | --- | --- | --- | --- | --- |
|  |  |  | **GMR** | **P-val** | **95%CI-L** | **95%CI-H** | **GMR** | **P-val** | **95%CI-L** | **95%CI-H** | **GMR** | **P-val** | **95%CI-L** | **95%CI-H** | **ESTIM** | **P-val** | **95%CI-L** | **95%CI-H** |
| **CHILD CHARACTERISTICS** | | | | | | | | | | | | | | | | | | |
| **Age (months)** | Age |  | 1.052 | **<0.001** | 1.043 | 1.061 | 1.035 | **<0.001** | 1.029 | 1.040 | 1.060 | **<0.001** | 1.047 | 1.073 | - 0.003 | **<0.001** | -0.004 | -0.002 |
|  | Age^2^ |  | 0.9994 | **<0.001** | 0.9993 | 0.9996 | 0.9996 | **<0.001** | 0.9995 | 0.9997 | 0.9993 | **<0.001** | 0.9991 | 0.9995 |  |  |  |  |
| **Sex** | Boys | 34 (56.7%) | 1 |  |  |  | 1 |  |  |  | 1 |  |  |  |  |  |  |  |
|  | Girls | 26 (43.3%) | 1.165 | 0.169 | 0.937 | 1.447 | 1.059 | 0.159 | 0.978 | 1.146 | 1.199 | **0.055** | 0.996 | 1.444 | 0.012 | 0.478 | -0.021 | 0.044 |
| **Delivery method** | Vaginal | 47(78.3%) | 1 |  |  |  | 1 |  |  |  | 1 |  |  |  |  |  |  |  |
|  | Caesarean | 13(21.7%) | 1.099 | 0.483 | 0.844 | 1.431 | 1.059 | 0.241 | 0.962 | 1.165 | 1.093 | 0.444 | 0.870 | 1.373 | -0.030 | 0.140 | -0.069 | 0.010 |
| **Birthweight (kg)** | Mean/SD | 3.33/.57 | 1.013 | 0.895 | 0.837 | 1.225 | 0.966 | 0.325 | 0.902 | 1.035 | 0.902 | 0.203 | 0.769 | 1.057 | -0.011 | 0.485 | -0.041 | 0.019 |
| **Birth order** | 1^st^ -4^th^ | 43 (71.7%) | 1 |  |  |  | 1 |  |  |  | 1 |  |  |  |  |  |  |  |
|  | >=5^th^ | 17 (28.3%) | 0.987 | 0.917 | 0.774 | 1.259 | 1.070 | **0.283** | 0.946 | 1.211 | 0.961 | 0.776 | 1.190 | 1.258 | -0.031 | **0.057** | -0.062 | 0.0008 |
|  | x Age |  |  |  |  |  | 0.996 | **0.039** | 0.993 | 0.999 |  |  |  |  |  |  |  |  |
| **Exclusive breastfeeding (m)** | Mean/SD | 4.12 / 2.17 | 1.048 | **0.038** | 1.003 | 1.095 | 1.004 | 0.600 | 0.988 | 1.019 | 1.001 | 0.944 | 0.965 | 1.039 | 0.002 | 0.601 | -0.006 | 0.010 |
| **Weaning age (m)** | Mean/SD | 6.22/1.81 | 1.022 | 0.283 | 0.982 | 1.064 | 1.015 | 0.123 | 0.996 | 1.034 | 1.032 | 0.178 | 0.986 | 1.081 | 0.003 | 0.476 | -0.006 | 0.013 |
| **Dietary patterns** |  |  |  |  |  |  |  |  |  |  |  |  |  |  |  |  |  |  |
| **‘Traditional’** | Low | 24(49.0%) | 1 |  |  |  | 1 |  |  |  | 1 |  |  |  |  |  |  |  |
|  | High | 25(51.0%) | 1.365 | **0.006** | 1.093 | 1.704 | 1.017 | 0.770 | 0.910 | 1.137 | 0.992 | 0.935 | 0.813 | 1.210 | -0.021 | 0.220 | -0.054 | 0.012 |
| **‘Breakfast’** | Low | 28(57.1%) | 1 |  |  |  | 1 |  |  |  | 1 |  |  |  |  |  |  |  |
|  | High | 21(42.9%) | 1.002 | 0.984 | 0.786 | 1.279 | 0.947 | 0.340 | 0.848 | 1.059 | 0.961 | 0.699 | 0.788 | 1.173 | -0.018 | 0.287 | -0.051 | 0.015 |
| **‘Vegetables and fats’** | Low | 19(38.8%) | 1 |  |  |  | 1 |  |  |  | 1 |  |  |  |  |  |  |  |
|  | High | 30(61.2%) | 1.171 | 0.202 | 0.919 | 1.491 | 1.043 | 0.456 | 0.934 | 1.165 | 1.078 | 0.461 | 0.883 | 1.315 | -0.041 | **0.014** | -0.073 | -0.008 |
| **‘Sweets’** | Low | 23(46.9%) | 1 |  |  |  | 1 |  |  |  | 1 |  |  |  |  |  |  |  |
|  | High | 26(52.1%) | 0.915 | 0.470 | 0.719 | 1.164 | 0.938 | 0.246 | 0.841 | 1.045 | 0.868 | 0.154 | 0.714 | 1.055 | 0.038 | **0.022** | 0.005 | 0.070 |
| **Unpasteurized milk (TV)** | Never | 5(8.3%) | 1 |  |  |  | 1 |  |  |  | 1 |  |  |  |  |  |  |  |
|  | Sometimes | 21(35.0%) | 1.125 | 0.204 | 0.938 | 1.348 | 1.065 | 0.220 | 0.963 | 1.177 | 0.982 | 0.905 | 0.730 | 1.321 | 0.046 | **0.057** | -0.001 | 0.094 |
|  | Frequently | 34(56.7%) | 1.227 | **0.041** | 1.008 | 1.493 | 1.161 | **0.008** | 1.039 | 1.297 | 0.925 | 0.716 | 0.608 | 1.408 | 0.040 | 0.115 | -0.010 | 0.090 |
|  | Sometimes× Age |  |  |  |  |  |  |  |  |  | 1.022 | **0.017** | 1.004 | 1.040 |  |  |  |  |
|  | Often × Age |  |  |  |  |  |  |  |  |  | 1.029 | **0.006** | 1.008 | 1.050 |  |  |  |  |
| **Day-care** | No | 52 (86.7%) | 1 |  |  |  | 1 |  |  |  | 1 |  |  |  |  |  |  |  |
|  | Yes | 8 (13.3%) | 1.120 | 0.365 | 0.876 | 1.432 | 0.994 | 0.916 | 0.894 | 1.106 | 1.009 | 0.941 | 0.787 | 1.295 | 0.002 | 0.933 | -0.045 | 0.048 |
| **Antibiotics (TV)** | No | 27(45.0 %) | 1 |  |  |  | 1 |  |  |  | 1 |  |  |  |  |  |  |  |
|  | Yes | 33(55.0%) | 0.994 | 0.937 | 0.855 | 1.155 | 0.974 | 0.489 | 0.904 | 1.049 | 0.977 | 0.787 | 0.828 | 1.154 | 0.001 | 0.905 | -0.017 | 0.019 |
| **Anthelmintics (TV)** | No | 8(13.3%) | 1 |  |  |  | 1 |  |  |  | 1 |  |  |  |  |  |  |  |
|  | Yes | 52(86.7%) | 0.939 | 0.431 | 0.803 | 1.098 | 1.011 | 0.819 | 0.923 | 1.106 | 0.996 | 0.975 | 0.801 | 1.239 | 0.010 | 0.615 | -0.030 | 0.050 |
| **Any STH (TV)** | No | 38(63.3%) | 1 |  |  |  | 1 |  |  |  | 1 |  |  |  |  |  |  |  |
|  | Yes | 22(37.7%) | 0.956 | 0.629 | 0.797 | 1.147 | 1.070 | 0.161 | 0.973 | 1.178 | 1.207 | 0.121 | 0.951 | 1.531 | -0.031 | 0.223 | -0.080 | 0.019 |
| ***A. lumbricoides* (TV)** | No | 41(68.3%) | 1 |  |  |  | 1 |  |  |  | 1 |  |  |  |  |  |  |  |
|  | Yes | 19(31.7%) | 0.828 | **0.055** | 0.682 | 1.004 | 1.033 | 0.549 | 0.929 | 1.148 | 1.108 | 0.434 | 0.856 | 1.434 | -0.031 | 0.265 | -0.086 | 0.024 |
| ***T. trichiura* (TV)** | No | 38(63.3%) | 1 |  |  |  | 1 |  |  |  | 1 |  |  |  |  |  |  |  |
|  | Yes | 12(36.7%) | 1.246 | **0.067** | 0.985 | 1.577 | 1.078 | 0.226 | 0.955 | 1.216 | 1.263 | 0.133 | 0.932 | 1.713 | -0.025 | 0.430 | -0.088 | 0.037 |
| **MATERNAL CHARACTERISTICS** | | | | | | | | | | | | | | | | | | |
| **Age (years)** | <=20 | 18 (30%) | 1 |  |  |  | 1 |  |  |  | 1 |  |  |  |  |  |  |  |
|  | >20 | 42(70%) | 1.194 | 0.130 | .949 | 1.503 | .983 | 0.698 | .903 | 1.070 | .944 | 0.574 | .773 | 1.153 | -.015 | 0.396 | -.048 | .019 |
| **Education** | Illiterate | 14 (23.3%) | 1 |  |  |  | 1 |  |  |  | 1 |  |  |  |  |  |  |  |
|  | Primary/Second | 46 (76.7%) | 0.934 | 0.621 | 0.714 | 1.223 | 1.099 | **0.063** | 0.995 | 1.215 | 1.250 | **0.069** | 0.983 | 1.591 | .142 | **0.001** | .060 | .224 |
|  | Prim/Sec × Age |  |  |  |  |  |  |  |  |  |  |  |  |  | -.004 | **0.004** | -.006 | -.001 |
| **Ethnicity** | Afro-Ecuadorian | 12 (20%) | 1 |  |  |  | 1 |  |  |  | 1 |  |  |  |  |  |  |  |
|  | Non-Afro-Ecuad. | 48 (80%) | 0.865 | 0.283 | 0.665 | 1.127 | 1.031 | 0.527 | 0.937 | 1.135 | 1.201 | 0.108 | 0.961 | 1.501 | 0.003 | 0.884 | -0.034 | 0.037 |
| **STH during pregnancy** | No | 31 (51.7%) | 1 |  |  |  | 1 |  |  |  | 1 |  |  |  |  |  |  |  |
|  | Yes | 29 (48.3%) | 0.863 | 0.176 | 0.698 | 1.068 | 0.926 | **0.047** | 0.859 | 0.999 | 0.870 | 0.135 | 0.725 | 1.044 | 0.007 | 0.675 | -0.024 | 0.038 |
| **Antibiotics during** | No | 27 (47.4%) | 1 |  |  |  | 1 |  |  |  | 1 |  |  |  |  |  |  |  |
| **pregnancy** | Yes | 30 (52.6%) | 1.028 | 0.803 | 0.826 | 1.281 | 0.962 | 0.348 | 0.886 | 1.044 | 0.951 | 0.616 | 0.781 | 1.157 | 0.006 | 0.774 | -0.032 | 0.043 |
| **SOCIO-ECONOMIC FACTORS** | | | | | | | | | | | | | | | | | | |
| **Socio-economic status** | Lower | 27 (45%) | 1 |  |  |  | 1 |  |  |  | 1 |  |  |  |  |  |  |  |
|  | Higher | 33 (55%) | 0.667 | **0.003** | 0.513 | 0.868 | 1.012 | 0.786 | 0.930 | 1.101 | 1.061 | 0.565 | 0.868 | 1.295 | .048 | **0.159** | -.019 | .116 |
|  | Higher × Age |  | 1.007 | **0.023** | 1.001 | 1.013 |  |  |  |  |  |  |  |  | -.002 | **0.007** | -.004 | -.001 |
| **Monthly income** | <1 basic salary | 54(90%) | 1 |  |  |  | 1 |  |  |  | 1 |  |  |  |  |  |  |  |
|  | >1 basic salary | 3(5%) | 1.005 | 0.984 | 0.628 | 1.606 | 0.958 | 0.577 | 0.825 | 1.113 | 0.985 | 0.929 | 0.698 | 1.389 | 0.003 | 0.915 | -.051 | .056 |
| **Material goods** | 0-2 | 42 (70%) | 1 |  |  |  | 1 |  |  |  | 1 |  |  |  |  |  |  |  |
|  | 3-4 | 18 (30%) | 0.827 | 0.101 | 0.659 | 1.038 | 1.011 | 0.784 | 0.932 | 1.097 | 1.045 | 0.650 | 0.863 | 1.266 | -0.017 | 0.301 | -0.049 | 0.015 |
| **HOUSEHOLD ENVIRONMENT** | | | | | | | | | | | | | | | | | | |
| **Residence** | Urban | 30 (50%) | 1 |  |  |  | 1 |  |  |  | 1 |  |  |  |  |  |  |  |
|  | Rural | 30 (50%) | 1.081 | 0.484 | 0.869 | 1.346 | 1.033 | 0.427 | 0.953 | 1.120 | 1.090 | 0.373 | 0.901 | 1.318 | 0.007 | 0.668 | -0.025 | 0.040 |
| **Crowding (at birth)** | <3 | 25 (41.7%) | 1 |  |  |  | 1 |  |  |  | 1 |  |  |  |  |  |  |  |
|  | >=3 | 35 (58.3%) | 1.299 | **0.040** | 1.012 | 1.668 | 0.962 | 0.326 | 0.889 | 1.040 | 0.867 | 0.127 | 0.721 | 1.041 | 0.014 | 0.410 | -0.019 | 0.046 |
|  | (>=3 vs. <3) × Age |  | 0.994 | **0.019** | 0.988 | 0.999 |  |  |  |  |  |  |  |  |  |  |  |  |
| **House construction** | Wood/Bamboo | 30(50.8%) | 1 |  |  |  | 1 |  |  |  | 1 |  |  |  |  |  |  |  |
| **(at birth)** | Cement/Brick | 29(49.2%) | 0.726 | **0.018** | 0.556 | 0.947 | 1.042 | 0.324 | 0.961 | 1.129 | 1.123 | 0.240 | 0.926 | 1.363 | -0.003 | 0.883 | -0.040 | 0.034 |
|  | × Age |  | 1.008 | **0.005** | 1.003 | 1.014 |  |  |  |  |  |  |  |  |  |  |  |  |
| **Potable water (TV)** | No | 45 (75%) | 1 |  |  |  | 1 |  |  |  | 1 |  |  |  |  |  |  |  |
|  | Yes | 15 (25%) | 1.069 | 0.587 | 0.840 | 1.361 | 1.023 | 0.600 | 0.939 | 1.115 | 1.002 | 0.983 | 0.817 | 1.229 | 0.001 | 0.944 | -0.032 | 0.035 |
| **Bathroom (TV)** | Latrine | 51 (85%) | 1 |  |  |  | 1 |  |  |  | 1 |  |  |  |  |  |  |  |
|  | WC | 9 (15%) | 1.133 | 0.172 | 0.947 | 1.355 | 0.977 | 0.616 | 0.894 | 1.069 | 0.933 | 0.518 | 0.757 | 1.150 | -0.025 | 0.244 | -0.067 | 0.017 |
| **Fridge (at birth)** | No | 32(56.1%) | 1 |  |  |  | 1 |  |  |  | 1 |  |  |  |  |  |  |  |
|  | Yes | 25(43.9%) | 0.963 | 0.741 | 0.769 | 1.206 | 1.062 | 0.128 | 0.983 | 1.148 | 1.112 | 0.259 | 0.924 | 1.338 | -0.019 | 0.243 | -0.050 | 0.013 |
| **Dog in house (TV)** | No | 57 (95%) | 1 |  |  |  | 1 |  |  |  | 1 |  |  |  |  |  |  |  |
|  | Yes | 3 (5%) | 1.423 | 0.153 | 0.877 | 2.308 | 0.956 | 0.556 | 0.823 | 1.111 | 0.906 | 0.577 | 0.642 | 1.280 | 0.030 | 0.273 | -0.024 | 0.085 |
|  | × Age |  | 0.988 | **0.011** | 0.978 | 0.997 |  |  |  |  |  |  |  |  |  |  |  |  |
| **Cat in house (TV)** | No | 52 (86.7%) | 1 |  |  |  | 1 |  |  |  | 1 |  |  |  |  |  |  |  |
|  | Yes | 8 (13.3%) | 1.124 | 0.470 | 0.818 | 1.545 | 0.968 | 0.584 | 0.860 | 1.089 | 0.934 | 0.623 | 0.712 | 1.226 | 0.002 | 0.934 | -0.048 | 0.052 |
| **Peri-domestic animals** |  |  |  |  |  |  |  |  |  |  |  |  |  |  |  |  |  |  |
| **Pigs (TV)** | No | 28(46.7%) | 1 |  |  |  | 1 |  |  |  | 1 |  |  |  |  |  |  |  |
|  | Yes | 32(53.3%) | 1.121 | 0.117 | 0.972 | 1.294 | 1.035 | 0.371 | 0.960 | 1.115 | 1.048 | 0.603 | 0.879 | 1.248 | 0.004 | 0.853 | -0.034 | 0.042 |
| **Cows (TV)** | No | 54(90%) | 1 |  |  |  | 1 |  |  |  | 1 |  |  |  |  |  |  |  |
|  | Yes | 6(10%) | 1.076 | 0.693 | 0.747 | 1.549 | 1.191 | **0.070** | 0.986 | 1.439 | 3.402 | **0.003** | 1.497 | 7.731 | -0.004 | 0.941 | -0.117 | 0.108 |
|  | × Age |  |  |  |  |  |  |  |  |  | 0.976 | **0.041** | 0.953 | 0.999 |  |  |  |  |
| **Horses (TV)** | No | 46(76.7%) | 1 |  |  |  | 1 |  |  |  | 1 |  |  |  |  |  |  |  |
|  | Yes | 14(23.3%) | 1.336 | **0.006** | 1.087 | 1.641 | 1.074 | 0.216 | 0.959 | 1.204 | 1.117 | 0.421 | 0.853 | 1.462 | 0.030 | 0.309 | -0.028 | 0.087 |
| **Agricultural exposure (TV)** | No | 33(55%) | 1 |  |  |  | 1 |  |  |  | 1 |  |  |  |  |  |  |  |
|  | Yes | 27(45%) | 1.034 | 0.724 | 0.858 | 1.247 | 1.097 | **0.038** | 1.005 | 1.197 | 1.248 | **0.076** | 0.977 | 1.594 | -0.033 | 0.263 | -0.092 | 0.025 |
| **Any STH** | No | 31 (51.7%) | 1 |  |  |  | 1 |  |  |  | 1 |  |  |  |  |  |  |  |
|  | Yes | 29 (48.3%) | 0.888 | 0.310 | 0.706 | 1.117 | 0.895 | **0.003** | 0.833 | 0.962 | 0.810 | **0.023** | 0.675 | 0.972 | -0.040 | 0.230 | -0.105 | 0.025 |
|  | × Age |  |  |  |  |  |  |  |  |  |  |  |  |  | 0.003 | **0.013** | 0.001 | 0.005 |

Table S2. Associations between age-adjusted estimates for alpha (Chao, Shannon, InvSimpson) and beta diversity measures and individual, maternal, and household exposures. Estimates for Chao, Shannon and InvSimpson represent geometric mean ratios (GMR) or fold changes for one unit increase in a continuous explanatory variable or for a level of a category against the baseline of a categorical explanatory variable. Estimates for beta (reflecting the change in the microbiota between two adjacent time points or rate of change in diversity) are minimally adjusted for the age of the first measurement. The estimates were obtained after fitting a mixed model to the longitudinal observations representing age-specific scores of these indices on log scale to comply with the models’ normality assumption. Polynomial terms for age were included as were the exposure variables as predictors. Interactions with age are presented only if significant. For beta diversity, if the explanatory variable is continuous, estimates represent monthly rate of change for one unit increase in that variable adjusted for the age at the first measurement, and if categorical estimates represent the difference in the monthly rate of change between the levels of the categorical variable and baseline level. House construction materials were traditional (wood and bamboo) or non-traditional (cement or brick). One basic salary (also known as canasta familiar básica [basic family basket]) was the amount estimated by the Institute of Censuses and Statistics in Ecuador (INEC) to be required to purchase a set of goods and services that are considered indispensable to satisfy the basic needs of a household of four members - this amount in 2008 was US$480 monthly. Missing data: dietary patterns (11); maternal antibiotics during pregnancy (3); fridge (3); house construction (1). Abbreviations: Estim – estimate. m – months; SD – standard deviation; CI- confidence interval; TV – time-varying; STH- soil-transmitted helminth; Afro – Afro-Ecuadorian; x variable – interaction effect.

| **Actinobacteria** |  | **Bacteroidetes** | | | | **Firmicutes** | | | | **Proteobacteria** | | | | **Verrucomicrobia** | | | | **Bacteria classified** | | | | **Bacteria unclassified** | | | |  |
| --- | --- | --- | --- | --- | --- | --- | --- | --- | --- | --- | --- | --- | --- | --- | --- | --- | --- | --- | --- | --- | --- | --- | --- | --- | --- | --- |
|  |  | **Estim** | **p-value** | **95%CI -L** | **95%CI -H** | **Estim** | **p-value** | **95%CI -L** | **95%CI -H** | **Estim** | **p-value** | **95%CI -L** | **95%CI -H** | **Estim** | **p-value** | **95%CI -L** | **95%CI -H** | **Estim** | **p-value** | **95%CI -L** | **95%CI -H** | **Estim** | **p-value** | **95%CI -L** | **95%CI -H** |  |
| **CHILD CHARACTERISTICS** | | | | | | | | | | | | | | | | | | | | | | | | | | |
| **Sex** | Girls vs Boys | -0.132 | 0.512 | -0.527 | 0.263 | 0.000 | 0.998 | -0.315 | 0.314 | -0.359 | 0.084 | -0.767 | 0.048 | -0.049 | 0.760 | -0.362 | 0.265 | 0.005 | 0.975 | -0.340 | 0.351 | -0.021 | 0.902 | -0.352 | 0.310 |  |
| **Delivery method** | C-sect vs Vaginal | 0.472 | 0.139 | -0.154 | 1.097 | 0.164 | 0.641 | -0.525 | 0.853 | 0.177 | 0.644 | -0.574 | 0.928 | 0.340 | 0.301 | -0.304 | 0.983 | 0.187 | 0.544 | -0.419 | 0.794 | -0.018 | 0.946 | -0.529 | 0.493 |  |
|  | x Age | **-0.025** | **0.035** | **-0.048** | **-0.002** | -0.010 | 0.215 | -0.026 | 0.006 | **-0.017** | **0.042** | **-0.034** | **-0.001** | **-0.020** | **0.024** | **-0.037** | **-0.003** | -0.010 | 0.220 | -0.025 | 0.006 | -0.004 | 0.480 | -0.017 | 0.008 |  |
| **Birthweight (kg)** | Mean/SD | -0.076 | 0.652 | -0.405 | 0.253 | -0.041 | 0.788 | -0.339 | 0.257 | 0.140 | 0.394 | -0.182 | 0.462 | 0.071 | 0.508 | -0.138 | 0.279 | 0.002 | 0.991 | -0.299 | 0.303 | 0.021 | 0.869 | -0.230 | 0.273 |  |
| **Birth order** | >=5^th^ vs. 1^st^ -4^th^ | 0.216 | 0.432 | -0.322 | 0.753 | 0.088 | 0.645 | -0.287 | 0.464 | 0.258 | 0.300 | -0.230 | 0.745 | 0.001 | 0.995 | -0.361 | 0.363 | 0.035 | 0.865 | -0.373 | 0.443 | 0.227 | 0.244 | -0.155 | 0.608 |  |
|  | x Age | 0.351 | 0.053 | -0.005 | 0.707 | 0.057 | 0.757 | -0.304 | 0.418 | 0.086 | 0.686 | -0.332 | 0.505 | -0.078 | 0.665 | -0.432 | 0.276 | 0.123 | 0.534 | -0.265 | 0.510 | 0.263 | 0.152 | -0.097 | 0.623 |  |
| **Excl. breastfeeding (m)** | Mean/SD | 0.016 | 0.690 | -0.061 | 0.093 | -0.024 | 0.471 | -0.087 | 0.040 | -0.008 | 0.858 | -0.091 | 0.076 | -0.006 | 0.859 | -0.072 | 0.060 | -0.034 | 0.302 | -0.098 | 0.030 | 0.007 | 0.832 | -0.060 | 0.075 |  |
| **Weaning age (m)** | Mean/SD | 0.058 | 0.474 | -0.100 | 0.215 | **0.089** | **0.024** | **0.012** | **0.166** | 0.048 | 0.449 | -0.077 | 0.173 | 0.062 | 0.076 | -0.006 | 0.131 | 0.075 | 0.146 | -0.026 | 0.175 | 0.082 | 0.073 | -0.008 | 0.171 |  |
| **Dietary patterns** |  |  |  |  |  |  |  |  |  |  |  |  |  |  |  |  |  |  |  |  |  |  |  |  |  |  |
| **‘Traditional’** | High vs. Low | 0.280 | 0.169 | -0.119 | 0.679 | -0.011 | 0.950 | -0.341 | 0.320 | 0.133 | 0.538 | -0.290 | 0.556 | 0.029 | 0.859 | -0.295 | 0.354 | -0.045 | 0.810 | -0.409 | 0.319 | 0.028 | 0.864 | -0.291 | 0.346 |  |
| **‘Breakfast’** | High vs. Low | -0.253 | 0.205 | -0.645 | 0.138 | -0.303 | 0.072 | -0.634 | 0.027 | -0.370 | 0.083 | -0.787 | 0.048 | -0.172 | 0.313 | -0.506 | 0.162 | -0.262 | 0.153 | -0.621 | 0.097 | 0.075 | 0.652 | -0.250 | 0.400 |  |
| **‘Vegetables and fats’** | High vs. Low | 0.241 | 0.239 | -0.160 | 0.642 | 0.085 | 0.602 | -0.235 | 0.406 | -0.057 | 0.791 | -0.481 | 0.367 | 0.100 | 0.541 | -0.220 | 0.421 | 0.106 | 0.559 | -0.250 | 0.462 | -0.120 | 0.445 | -0.427 | 0.188 |  |
| **‘Sweets’** | High vs. Low | 0.063 | 0.764 | -0.346 | 0.471 | -0.106 | 0.522 | -0.430 | 0.218 | 0.046 | 0.831 | -0.373 | 0.465 | -0.147 | 0.356 | -0.459 | 0.165 | -0.132 | 0.472 | -0.492 | 0.228 | -0.075 | 0.643 | -0.392 | 0.242 |  |
| **Unpasteurized milk (TV)** | Ever vs. Never | -0.247 | 0.350 | -0.764 | 0.271 | 0.369 | 0.151 | -0.135 | 0.873 | 0.053 | 0.844 | -0.479 | 0.586 | 0.154 | 0.477 | -0.270 | 0.578 | -0.010 | 0.964 | -0.459 | 0.439 | -0.108 | 0.638 | -0.559 | 0.342 |  |
| **Day-care** | Yes vs. No | 0.268 | 0.267 | -0.205 | 0.742 | 0.212 | 0.203 | -0.114 | 0.538 | 0.363 | 0.062 | -0.019 | 0.745 | 0.270 | 0.156 | -0.103 | 0.644 | **0.459** | **0.010** | **0.107** | **0.810** | 0.145 | 0.463 | -0.243 | 0.534 |  |
| **Antibiotics (TV)** | Yes vs. No | -0.124 | 0.134 | -0.287 | 0.038 | -0.020 | 0.780 | -0.163 | 0.123 | -0.074 | 0.402 | -0.248 | 0.099 | 0.025 | 0.661 | -0.088 | 0.138 | -0.043 | 0.471 | -0.161 | 0.075 | -0.095 | 0.269 | -0.265 | 0.074 |  |
| **Anthelmintics (TV)** | Yes vs. No | -0.099 | 0.855 | -1.159 | 0.962 | -0.031 | 0.868 | -0.395 | 0.333 | -0.043 | 0.865 | -0.537 | 0.452 | -0.090 | 0.777 | -0.717 | 0.537 | -0.015 | 0.960 | -0.590 | 0.560 | -0.056 | 0.837 | -0.593 | 0.480 |  |
| **Any STH (TV)** | Yes vs. No | 0.142 | 0.624 | -0.426 | 0.710 | -0.183 | 0.387 | -0.597 | 0.232 | -0.092 | 0.751 | -0.657 | 0.474 | -0.288 | 0.142 | -0.673 | 0.096 | 0.062 | 0.789 | -0.390 | 0.514 | -0.018 | 0.937 | -0.472 | 0.436 |  |
| ***A. lumbricoides* (TV)** | Yes vs. No | -0.050 | 0.826 | -0.491 | 0.392 | -0.050 | 0.826 | -0.491 | 0.392 | 0.181 | 0.547 | -0.410 | 0.772 | -0.153 | 0.450 | -0.548 | 0.243 | 0.298 | 0.221 | -0.180 | 0.776 | 0.021 | 0.938 | -0.522 | 0.565 |  |
| ***T. trichiura* (TV)** | Yes vs. No | -.969 | 0.069 | -2.013 | 0.075 | **-1.537** | **0.005** | **-2.618** | **-0.457** | **-1.617** | **0.001** | **-2.537** | **-0.697** | **-1.493** | **0.002** | **-2.417** | **-0.570** | -1.024 | 0.054 | -2.063 | 0.016 | -1.191 | 0.065 | -2.454 | 0.073 |  |
|  | × Age | .028 | 0.077 | -0.003 | 0.060 | **0.035** | **0.019** | **0.006** | **0.063** | **0.037** | **0.005** | **0.011** | **0.062** | **.036** | **0.018** | **0.006** | **0.066** | 0.027 | 0.088 | -0.004 | 0.057 | 0.034 | 0.051 | 0.000 | 0.069 |  |
| **MATERNAL CHARACTERISTICS** | | | | | | | | | | | | | | | | | | | | | | | | | | |
| **Age (years)** | >20 vs. <=20 | 0.128 | 0.528 | -0.269 | 0.524 | -0.004 | 0.984 | -0.389 | 0.381 | -0.074 | 0.742 | -0.515 | 0.366 | **-0.357** | **0.023** | **-0.665** | **-0.050** | -0.022 | 0.912 | -0.410 | 0.367 | 0.144 | 0.434 | -0.216 | 0.503 |  |
| **Education** | Primary/Second vs Illiterate | -0.011 | 0.952 | -0.386 | 0.363 | -0.003 | 0.988 | -0.383 | 0.377 | 0.053 | 0.796 | -0.349 | 0.455 | 0.001 | 0.997 | -0.337 | 0.338 | 0.034 | 0.843 | -0.300 | 0.367 | -0.094 | 0.610 | -0.456 | 0.268 |  |
| **Ethnicity** | Non Afro- vs Afro | 0.233 | 0.193 | -0.118 | 0.584 | 0.222 | 0.204 | -0.121 | 0.566 | **0.441** | **0.049** | **0.002** | **0.879** | 0.317 | 0.130 | -0.094 | 0.729 | 0.209 | 0.245 | -0.143 | 0.560 | -0.082 | 0.689 | -0.484 | 0.320 |  |
| **STH during pregnancy** | Yes vs. No | 0.095 | 0.616 | -0.277 | 0.468 | -0.087 | 0.594 | -0.410 | 0.235 | -0.149 | 0.453 | -0.538 | 0.240 | -0.212 | 0.166 | -0.513 | 0.088 | 0.019 | 0.912 | -0.311 | 0.348 | 0.050 | 0.751 | -0.260 | 0.360 |  |
| **Antibiotics during pregnancy** | Yes vs. No | 0.015 | 0.937 | -0.351 | 0.381 | 0.049 | 0.755 | -0.258 | 0.355 | -0.074 | 0.714 | -0.469 | 0.321 | -0.154 | 0.321 | -0.459 | 0.150 | 0.056 | 0.735 | -0.268 | 0.379 | 0.088 | 0.585 | -0.228 | 0.404 |  |
| **SOCIO-ECONOMIC FACTORS** | | | | | | | | | | | | | | | | | | | | | | | | | | |
| **Socio-economic status** | Higher vs. Lower | 0.005 | 0.984 | -0.505 | 0.515 | 0.182 | 0.314 | -0.172 | 0.535 | -0.137 | 0.539 | -0.572 | 0.299 | 0.033 | 0.837 | -0.281 | 0.347 | 0.105 | 0.585 | -0.270 | 0.479 | 0.095 | 0.618 | -0.277 | 0.466 |  |
| **Monthly income** | >1 vs. <1  basic salary | -0.066 | 0.822 | -0.637 | 0.506 | -0.306 | 0.442 | -1.085 | 0.473 | -0.683 | **<0.001** | -1.051 | -0.315 | -0.407 | **0.003** | -0.675 | -0.140 | -0.461 | 0.078 | -0.974 | 0.051 | -0.344 | 0.108 | -0.765 | 0.076 |  |
| **Material goods** | 3-4 vs.0-2 | -0.208 | 0.269 | -0.576 | 0.160 | -0.097 | 0.549 | -0.416 | 0.221 | -0.300 | 0.120 | -0.678 | 0.078 | 0.018 | 0.919 | -0.328 | 0.364 | -0.245 | 0.134 | -0.564 | 0.075 | -0.074 | 0.648 | -0.394 | 0.245 |  |
| **HOUSEHOLD ENVIRONMENT** | | | | | | | | | | | | | | | | | | | | | | | | | | |
| **Residence** | Rural vs Urban | -0.016 | 0.939 | -0.420 | 0.388 | -0.056 | 0.731 | -0.373 | 0.261 | -0.162 | 0.467 | -0.597 | 0.274 | -0.116 | 0.456 | -0.422 | 0.189 | -0.061 | 0.737 | -0.415 | 0.293 | -0.098 | 0.560 | -0.428 | 0.232 |  |
| **Crowding (at birth)** | >=3 vs. <3 | 0.297 | 0.107 | -0.064 | 0.659 | 0.208 | 0.170 | -0.089 | 0.504 | 0.267 | 0.168 | -0.113 | 0.646 | -0.016 | 0.917 | -0.309 | 0.278 | 0.150 | 0.363 | -0.173 | 0.473 | 0.260 | 0.091 | -0.042 | 0.562 |  |
| **House construction**  **(at birth)** | Cement/Brick vs. Wood/Bamboo | -0.336 | 0.121 | -0.761 | 0.089 | -0.020 | 0.902 | -0.345 | 0.304 | -0.430 | **0.025** | -0.806 | -0.055 | -0.039 | 0.812 | -0.361 | 0.282 | -0.108 | 0.517 | -0.434 | 0.218 | -0.113 | 0.522 | -0.459 | 0.233 |  |
| **Potable water (TV)** | Yes | -0.285 | 0.083 | -0.607 | 0.038 | -0.145 | 0.390 | -0.474 | 0.185 | -0.224 | 0.219 | -0.582 | 0.134 | -0.253 | 0.079 | -0.535 | 0.029 | -0.169 | 0.343 | -0.519 | 0.180 | -0.151 | 0.321 | -0.449 | 0.147 |  |
| **Bathroom (TV)** | WC vs. Latrine | -0.094 | 0.628 | -0.473 | 0.285 | 0.115 | 0.496 | -0.215 | 0.445 | 0.266 | 0.067 | -0.018 | 0.550 | 0.124 | 0.449 | -0.196 | 0.443 | 0.215 | 0.335 | -0.222 | 0.651 | 0.166 | 0.345 | -0.179 | 0.511 |  |
| **Fridge (at birth)** | Yes vs. No | 0.134 | 0.498 | -0.253 | 0.522 | 0.165 | 0.310 | -0.153 | 0.482 | -0.013 | 0.947 | -0.400 | 0.373 | 0.168 | 0.279 | -0.136 | 0.471 | 0.115 | 0.501 | -0.220 | 0.449 | 0.177 | 0.272 | -0.139 | 0.493 |  |
| **Dog in house (TV)** | Yes vs. No | 0.244 | 0.608 | -0.687 | 1.174 | 0.257 | 0.456 | -0.419 | 0.934 | -0.059 | 0.923 | -1.257 | 1.138 | -0.034 | 0.928 | -0.772 | 0.704 | 0.195 | 0.655 | -0.660 | 1.049 | 0.398 | 0.182 | -0.187 | 0.984 |  |
| **Cat in house (TV)** | Yes vs. No | -0.413 | **0.026** | -0.776 | -0.050 | -0.196 | 0.443 | -0.698 | 0.305 | -0.374 | 0.251 | -1.013 | 0.265 | -0.237 | 0.281 | -0.667 | 0.193 | -0.336 | 0.150 | -0.794 | 0.122 | -0.040 | 0.860 | -0.484 | 0.404 |  |
| **Peri-domestic animals** |  |  |  |  |  |  |  |  |  |  |  |  |  |  |  |  |  |  |  |  |  |  |  |  |  |  |
| **Pigs (TV)** | Yes vs. No | -0.318 | 0.118 | -0.717 | 0.081 | **-0.419** | **0.013** | **-0.750** | **-0.088** | -0.349 | 0.109 | -0.776 | 0.077 | **-0.333** | **0.033** | **-0.639** | **-0.028** | **-0.398** | **0.035** | **-0.768** | **-0.027** | -0.174 | 0.308 | -0.510 | 0.161 |  |
| **Cows (TV)** | Yes vs. No | 0.173 | 0.615 | -0.503 | 0.849 | 0.230 | 0.195 | -0.118 | 0.577 | -0.321 | 0.115 | -0.721 | 0.079 | **0.614** | **0.002** | **0.222** | **1.005** | 0.330 | 0.252 | -0.235 | 0.895 | 0.153 | 0.320 | -0.148 | 0.455 |  |
| **Horses (TV)** | Yes vs. No | 0.246 | 0.343 | -0.263 | 0.756 | 0.036 | 0.841 | -0.318 | 0.390 | 0.035 | 0.877 | -0.406 | 0.475 | 0.213 | 0.239 | -0.141 | 0.567 | 0.069 | 0.752 | -0.358 | 0.496 | 0.363 | 0.167 | -0.152 | 0.878 |  |
| **Agricultural exposure (TV)** | Yes vs. No | -0.307 | 0.165 | -0.741 | 0.126 | -0.030 | 0.868 | -0.386 | 0.325 | -0.375 | 0.189 | -0.934 | 0.185 | -0.085 | 0.623 | -0.422 | 0.253 | -0.133 | 0.478 | -0.500 | 0.234 | -0.191 | 0.406 | -0.641 | 0.260 |  |
| **Any STH** | Yes vs. No | -0.436 | 0.101 | -0.958 | 0.086 | -0.463 | 0.119 | -1.045 | 0.119 | -0.375 | 0.199 | -0.948 | 0.198 | -0.357 | 0.123 | -0.811 | 0.097 | -0.372 | 0.125 | -0.848 | 0.103 | -0.428 | 0.103 | -0.943 | 0.087 |  |
|  | × Age | 0.015 | 0.118 | -0.004 | 0.034 | 0.013 | 0.085 | -0.002 | 0.028 | 0.017 | 0.054 | 0.000 | 0.034 | 0.004 | 0.671 | -0.013 | 0.020 | **0.015** | **0.023** | **0.002** | **0.028** | 0.008 | 0.241 | -0.005 | 0.021 |  |

Table S3. Associations between individual, maternal, and household exposures and phylum composition as a multivariate outcome consisting of age-specific proportions (Figure 2). Results were inferred using a Dirichlet-multinomial fit for the multivariate outcome representing the Phylum proportions across ages, namely Actinobacteria, Bacteroidetes, Firmicutes, Proteobacteria, Verrucomicrobia, Bacteria classified (Figure 2) and Bacteria unclassified. The standard errors and p-values account for the longitudinal features of the data and the estimates are adjusted for age (polynomial terms of powers up to 5). The estimates are relative ratios (log scale) and measure the age-adjusted effect of each explanatory variable on the relative abundance in the multivariate phylum components with Actinobacteria used as reference phylum. House construction materials were traditional (wood and bamboo) or non-traditional (cement or brick). One basic salary (also known as canasta familiar básica [basic family basket]) was the amount estimated by the Institute of Censuses and Statistics in Ecuador (INEC) to be required to purchase a set of goods and services that are considered indispensable to satisfy the basic needs of a household of four members - this amount in 2008 was US$480 monthly. Abbreviations: Estim. – estimate; CI- confidence interval; TV – time-varying; STH- soil-transmitted helminth; Afro – Afro-Ecuadorian. Variables were measured at birth (birth) or periodically during childhood (TV – time-varying); x variable – interaction effect.

**Summary of significant findings (i.e. P<0.05) in Table S4**. In the results below, we have back-transformed estimates to provide relative ratios (RR) of abundance with Actinobacteria used as reference group. The RRs represent overall effects during follow-up.

1. The presence of a cat in the household was associated (p=0.026) with a shift in phylum distribution, as indicated by the relative ratio of Bacteroidetes to Actinobacteria (RR = 0.661, 95% CI [0.460, 0.950]). This finding suggests children with household cats compared to those without had a relatively smaller proportion of Bacteroidetes relative to Actinobacteria.
2. A later age of weaning was associated with a higher proportion of Firmicutes relative to Actinobacteria. Specifically, each additional month of (later) weaning corresponded to an increase in the Firmicutes-to-Actinobacteria ratio (RR = 1.093, 95% CI [1.012, 1.180]) and consequently altered the overall distribution of phyla.
3. Children living in households with pigs compared to those without, had lower ratios of Firmicutes (RR = 0.658 (95% CI 0.473-0.916, p=0.013), Verrucomicrobia (RR = 0.716, 95% CI 0.528-0.973, p=0.033), and classified bacteria (RR = 0.672, 95% CI 0.464-0.973, p=0.035) relative to Actinobacteria.
4. Infection with *Trichuris* was significantly associated with shifts in phylum distribution, as indicated by lower ratios of Firmicutes (p=0.005), Verrucomicrobia (p=0.002), and classified bacteria (p=0.054) relative to Actinobacteria. The corresponding risk ratios were RR = 0.215 (95% CI [0.073, 0.633]), RR = 0.225 (95% CI [0.089, 0.566]), and RR = 0.359 (95% CI [0.127, 1.016]). These shifts exhibited age-related dynamics, with relative abundance ratios increasing monthly by RR = 1.035 (95% CI [1.006, 1.070]), RR = 1.037 (95% CI [1.006, 1.068]), and RR = 1.027 (95% CI [0.996, 1.059]), respectively.
5. Children born to non-Afro-Ecuadorian women were associated with a higher abundance of Verrucomicrobia relative to Actinobacteria (RR = 1.554, 95% CI [1.002, 2.410], p=0.049).
6. Higher monthly household income was associated with a lower abundance of Proteobacteria relative to Actinobacteria (p<0.001) and Verrucomicrobia relative to Actinobacteria (p=0.003) compared to lower income households. The corresponding risk ratios were RR = 0.505 (95% CI [0.349, 0.730]) and RR = 0.665 (95% CI [0.509, 0.869]), respectively.
7. Living in a house constricted with cement/brick was associated with a lower abundance of Verrucomicrobia relative to Actinobacteria (p=0.025, RR = 0.650, 95% CI [0.249, 0.946]).
8. The presence of cows around the household was associated with a higher abundance of Verrucomicrobia relative to Actinobacteria (p=0.002, RR = 1.847, 95% CI [0.446, 2.731]) compared to households without cows.
9. Soil-transmitted helminth (STH) infections in household members were significantly associated with shifts in phylum distribution exhibiting age-related dynamics (p=0.023). The relative abundance ratio of classified bacteria to Actinobacteria increased with age by RR = 1.014 (95% CI [1.002, 1.028]) per month.

| ***PREVOTELLA* TO *BACTEROIDES* RATIO** | | | | | |
| --- | --- | --- | --- | --- | --- |
| **Variable** | **Summary / category** | **Estimate** | **P-value** | **95%CI Low** | **95%CI High** |
| **CHILD CHARACTERISTICS** | | | | | |
| Age (months) | Age | 1.175 | **<.001** | 1.095 | 1.260 |
|  | Age^2^ | 0.9987 | 0.017 | .9976 | .9998 |
| Sex | Girls vs. Boys | 4.427 | 0.069 | .892 | 21.976 |
|  | (Girls vs. Boys) × Age | 0.957 | **0.035** | 0.919 | 0.996 |
| Birth order (birth) | 3^rd^ -4^th^ vs. 1^st^ -2^nd^ | 0.604 | 0.483 | 0.148 | 2.470 |
|  | > = 5^th^ vs. 1^st^ -2^nd^ | 2.274 | 0.258 | 0.548 | 9.443 |
| Birth order | > = 3^rd^ vs. 1^st^ -2^nd^ | 1.176 | 0.792 | 0.353 | 3.916 |
| Birthweight (kg) | The effect of 1 kg | 0.643 | 0.413 | 0.223 | 1.852 |
| Exclusive breastfeed age | Effect of one month | 1.041 | 0.781 | 0.786 | 1.379 |
| Age starting family diet | Effect of one month | 1.037 | 0.843 | 0.725 | 1.483 |
| Traditional | High vs. Low | 2.132 | 0.223 | 0.632 | 7.197 |
| Breakfast | High vs. Low | 0.804 | 0.728 | 0.235 | 2.747 |
| Vegetable fat | High vs. Low | 0.637 | 0.471 | 0.187 | 2.172 |
| Sweets | High vs. Low | 2.389 | 0.153 | 0.723 | 7.899 |
| Unpasteurized milk consumption (TV) | Sometimes vs. None | 0.476 | 0.351 | 0.100 | 2.264 |
|  | Often vs. None | 0.799 | 0.789 | 0.155 | 4.117 |
| Unpasteurized milk (binary) | Any vs None | 1.043 | 0.949 | 0.282 | 3.854 |
| Antibiotics (TV) | Yes vs. No | 0.414 | 0.108 | 0.142 | 1.213 |
| Antiparasitic treatments (TV) | Yes vs. No | 1.704 | 0.394 | 0.500 | 5.802 |
| Daily care to 36 months(TV) | Yes vs. No | 2.711 | 0.225 | 0.541 | 13.589 |
| STH (TV) | Yes vs. No | 3.963 | **0.033** | 1.115 | 14.088 |
| Ascaris (TV) | Yes vs. No | 3.608 | **0.075** | 0.879 | 14.817 |
| Trichuris (TV) | Yes vs. No | 4.947 | **0.054** | 0.971 | 25.197 |
| **MATERNAL CHARACTERISTICS** | | | | | |
| Age | 21-29 vs. <=20 | 1.871 | 0.387 | 0.452 | 7.738 |
|  | >=30 vs. <=20 | 0.967 | 0.967 | 0.200 | 4.681 |
| Age (binary) | >20 vs. <=20 | 1.100 | 0.887 | 0.297 | 4.070 |
| Education (birth) | Primary vs. Illiterate | 1.010 | 0.990 | 0.210 | 4.855 |
|  | Secondary vs. Illiterate | 0.282 | 0.177 | 0.045 | 1.772 |
| Education (binary) | Primary/Secondary vs. Illiterate | 0.723 | 0.685 | 0.150 | 3.482 |
| Ethnicity | Afro vs. Non Afro | 1.077 | 0.922 | 0.247 | 4.702 |
| STH at birth | Yes vs. No | 4.218 | **0.011** | 1.382 | 12.879 |
| Birth delivery | Caesarean vs. Vaginal | 0.828 | 0.802 | 0.190 | 3.615 |
| Maternal antibiotics during pregnancy | Yes vs. No | 0.274 | 0.115 | .055 | 1.367 |
|  | (Yes vs. No) × Age | 1.053 | **0.011** | 1.012 | 1.097 |
| **SOCIO-ECONOMIC CHARACTERISTICS** | | | | | |
| Soc-economic status (birth) | Medium vs. Low | 0.062 | **0.004** | 0.009 | 0.417 |
|  | High vs. Low | 0.092 | **0.015** | 0.013 | 0.625 |
|  | (Medium vs. Low) × Age | 1.078 | **0.004** | 1.025 | 1.134 |
|  | (High vs. Low) × Age | 1.053 | **0.046** | 1.001 | 1.108 |
| Soc-economic status (binary) | High/Medium vs. Low | 0.085 | **0.008** | 0.014 | 0.521 |
|  | (High/Medium vs. Low) × Age | 1.072 | **0.007** | 1.001 | 1.142 |
| Monthly income (birth) | Effect of 100$ | 0.837 | 0.467 | 0.517 | 1.354 |
| Monthly income (basic salary) | > 1 Basic vs. < 1 Basic | 0.345 | 0.380 | 0.032 | 3.721 |
| **HOUSEHOLD ENVIRONMENT** | | | | | |
| Residence (birth) | Rural vs. Urban | 3.206 | **0.052** | 0.992 | 10.363 |
| Crowding (birth) | >=3 vs. <3 per room | 1.912 | 0.289 | 0.577 | 6.337 |
| Crowding at birth | Increment by 1/room | 1.108 | 0.441 | 0.854 | 1.437 |
| Children house (TV) | Increment by 1/house | 1.160 | 0.434 | 0.800 | 1.682 |
| House Construction | Wood/Bamboo vs. Cement/Brick | 0.356 | 0.105 | 0.102 | 1.241 |
| Potable Water (birth) | Yes vs. No | 0.885 | 0.856 | 0.239 | 3.285 |
| Fridge house (birth) | Yes vs. No | .439 | 0.176 | 0.133 | 1.446 |
| Material goods (birth) | 3-4 vs. 0-2 | 0.238 | **0.018** | 0.073 | 0.781 |
| Bathroom (TV) | Yes vs. No | 0.265 | **0.038** | 0.076 | 0.932 |
| Dog in house (TV) | Yes vs. No | 1.735 | 0.642 | 0.170 | 17.683 |
| Cat in house (TV) | Yes vs. No | 0.331 | 0.224 | 0.056 | 1.967 |
| Pet in house (TV) | Yes vs. No | 0.385 | 0.236 | 0.080 | 1.865 |
| Pigs outside (at birth) | Yes vs. No | 1.738 | 0.405 | 0.474 | 6.375 |
| Pigs inside (TV) | Yes vs. No | 2.025 | 0.179 | 0.724 | 5.663 |
| Farm animals (TV) | Yes vs. No | 1.252 | 0.389 | 0.751 | 2.085 |
| Chick (TV) | Yes vs. No | 2.546 | 0.085 | 0.880 | 7.366 |
| Cows (TV) | Yes vs. No | 0.267 | 0.323 | 0.020 | 3.656 |
| Horse (TV) | Yes vs. No | 2.846 | 0.179 | 0.620 | 13.069 |
| Donkey (TV) | Yes vs. No | 1.892 | 0.511 | 0.282 | 12.690 |
| Mules (TV) | Yes vs. No | 0.604 | 0.582 | 0.100 | 3.639 |
| Any horse (TV) | 1 vs. None | 2.320 | 0.357 | 0.387 | 13.896 |
|  | >=2 vs. None | 1.978 | 0.483 | 0.294 | 13.326 |
| Agricultural exposure (birth) | Yes vs. No | 8.974 | **0.005** | 1.949 | 41.323 |
|  | (Yes vs. No) × Age | 0.959 | **0.036** | 0.923 | 0.997 |
| Agricultural exposure (TV) | Yes vs. No | 5.601 | 0.095 | 0.742 | 42.274 |
|  | (Yes vs. No) × Age | 0.949 | **0.045** | 0.902 | 0.999 |
| STH any house | Yes vs. No | 2.796 | 0.102 | 0.815 | 9.587 |

Table S4. Associations between individual, maternal, and household exposures and ratio of *Prevotella* to *Bacteriodetes* (log scale) using data from 60 children. Estimates were obtained using a mixed model to the longitudinal observations representing age-specific ratios of *Prevotella* to *Bacteriodetes*. House construction materials were traditional (wood and bamboo) or non-traditional (cement or brick). One basic salary (also known as canasta familiar básica [basic family basket]) was the amount estimated by the Institute of Censuses and Statistics in Ecuador (INEC) to be required to purchase a set of goods and services that are considered indispensable to satisfy the basic needs of a household of four members - this amount in 2008 was US$480 monthly. Abbreviations: Estim. – estimate; CI- confidence interval; TV – time-varying; STH- soil-transmitted helminth; Afro – Afro-Ecuadorian; x variable – interaction effect. Variables were measured at birth (birth) or periodically during childhood (TV – time-varying).


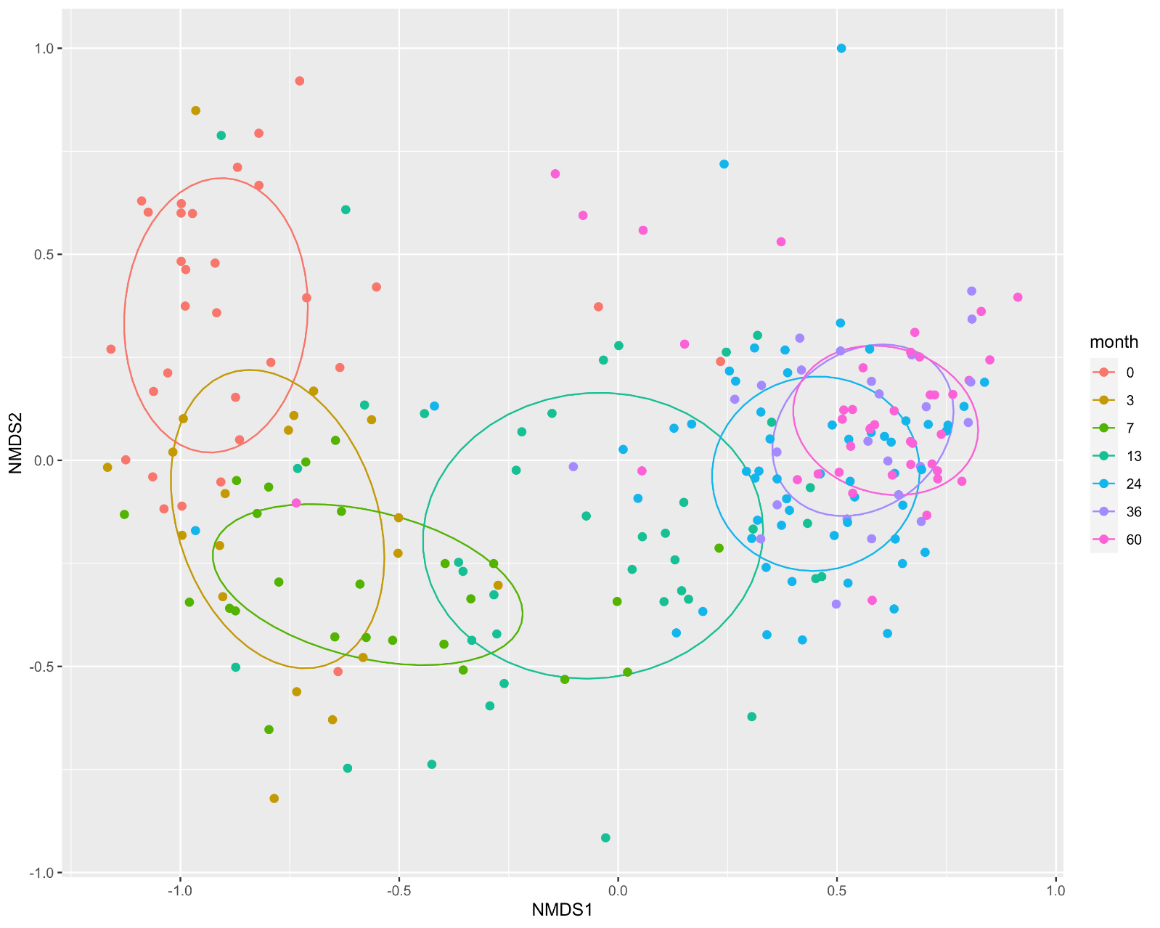


Figure S1. Exploratory NMDS Bray-Curtis-derived scatter plot for distributions of all analyzed samples by age using data from 60 children. Ellipses represent the mean of variation within each age group. This analysis is provided for the purpose of visualization only.

Figure S2. Individual exposures associated with significant age-dependent Chao scores (log scale) using data from 60 children. The graphs show the predicted trajectories across the whole cohort obtained after fitting a mixed model to the longitudinal observations representing age-specific Chao scores on log scale and including the categorical variables representing exposures in the set of predictors. Only exposures which significantly predicted age-dependent Chao scores (log scale) are shown. Abbreviations: Med – medium; 3/bedr – 3 household members per sleeping room; Socio-econ – socio-economic.

Figure S3. Individual exposures associated with significant age-dependent Shannon scores (log scale) in 60 children. The graphs show the predicted trajectories across the whole cohort obtained after fitting a mixed model to the longitudinal observations representing age-specific Shannon scores on log scale and including the categorical variables representing exposures in the set of predictors. Only exposures which significantly predicted age-dependent Shannon scores (log scale) are shown. Abbreviation: STH - soil-transmitted helminth.

Figure S4. Individual exposures associated with significant age-dependent changes in InvSimpson scores (log scale) using data from 60 children. The curves are the predicted trajectories across the whole cohort obtained after fitting a mixed model to the longitudinal observations representing age-specific InvSimpson scores on log scale and including the categorical variables representing exposures in the set of predictors. Only exposures which significantly predicted age-dependent InvSimpson scores (log scale) are shown. Abbreviations: cons - consumption; STH - soil-transmitted helminth.

Figure S5. Individual exposures associated with significant monthly changes in beta diversity score (log scale) using data from 60 children, adjusted for age at first measurement. Predictions are made for birth as age when the sampling started. The curves are the predicted trajectories across the cohort cohort obtained after fitting a mixed model to the longitudinal observations representing age-specific Beta scores on log scale and including the categorical variables representing exposures in the set of predictors. Only exposures which significantly predicted age-dependent Beta scores (log scale) are shown. Abbreviation: STH- soil-transmitted helminth.

Figure S6 – Individual exposures associated with significant age-dependent *Prevotella/Bacteroidetes* ratio (log scale) using data from 60 children. The curves are the predicted trajectories across the whole cohort obtained after fitting a mixed model to the longitudinal observations representing age-specific *Prevotella/Bacteroidetes* ratios on log scale and including the categorical variables representing exposures in the set of predictors. Only exposures which significantly predicted age-dependent age-dependent *Prevotella/Bacteroidetes* ratios (log scale) are shown. Abbreviations: STH- soil-transmitted helminth; TV – time-varying variable; social – socio-economic
